# Creatine and Beta‐Alanine Supplementation for Endurance Athletes: Ergogenic Aids for Reaching the Podium?

**DOI:** 10.1155/tsm2/8504414

**Published:** 2026-10-29

**Authors:** Felipe Ribeiro, Scott C. Forbes, Darren G. Candow, Erico Caperuto, Chad Kerksick, Fabio Santos Lira, Antonio Herbert Lancha, José C. Rosa Neto

**Affiliations:** ^1^ Department of Cell Biology and Developmental Institute of Biomedical Sciences University of São Paulo São Paulo São Paulo Brazil usp.br; ^2^ Department of Physical Education Studies Faculty of Education Brandon University Brandon Manitoba Canada brandonu.ca; ^3^ Faculty of Kinesiology and Health Studies University of Regina Regina Saskatchewan Canada uregina.ca; ^4^ University São Judas Tadeu São Paulo Brazil; ^5^ Mackenzie Presbyterian University São Paulo Brazil mackenzie.br; ^6^ College of Science, Technology, and Health Lindenwood University St. Charles Missouri USA lindenwood.edu; ^7^ Department of Physical Education Universidade Estadual Paulista (UNESP) São Paulo Brazil unesp.br; ^8^ Faculty of Medicine University of São Paulo São Paulo São Paulo Brazil usp.br

**Keywords:** beta-alanine, buffering capacity, creatine, endurance performance, fatigue resistance, muscle carnosine, sports nutrition, sprint finish

## Abstract

Endurance sports are characterized mainly by prolonged exercise demands. Although endurance performance is primarily supported by aerobic metabolism, anaerobic energy pathways, buffering capacity, neuromuscular resilience, and the ability to tolerate fatigue during decisive moments of a race are also relevant to competitive success. In this context, creatine and beta‐alanine supplementation are two ergogenic strategies purported to enhance endurance performance. This review critically examines the physiological rationale and current evidence regarding creatine supplementation, beta‐alanine supplementation, and their cosupplementation in endurance sports. The available literature suggests that beta‐alanine may improve performance in exercise tasks performed in the aerobic–anaerobic transition zone, through increased skeletal muscle carnosine content and enhanced intracellular buffering capacity. Creatine supplementation may support endurance performance by increasing phosphocreatine availability, improving glycogen resynthesis and buffering capacity, attenuating exercise‐induced inflammation and muscle damage, preserving fast‐twitch muscle characteristics, and enhancing repeated sprint ability or late‐race power output. However, evidence supporting improvements in classical markers of aerobic performance, such as VO_2_max, lactate threshold, and time‐to‐exhaustion, remains inconsistent, and the available evidence has been derived from untrained or recreationally active individuals, limiting direct translation to highly trained endurance athletes. Therefore, the practical relevance of these supplements appears to depend on the athlete’s training phase, event characteristics (e.g., weight‐bearing or not), competitive goal, and performance level. Creatine and beta‐alanine should not be viewed as universal ergogenic aids for endurance athletes, but rather as targeted nutritional strategies that may be particularly useful in race contexts where repeated high‐intensity efforts, fatigue resistance, and decisive finishing actions influence performance.

## 1. Introduction

Endurance sports, particularly running a half‐marathon or marathon, road cycling, and triathlon, encompass sports events lasting 30 min or longer and imposing multifactorial physiological demands that challenge multiple regulatory systems simultaneously, as these competitions can last one to seventeen hours [1]. Performance in these disciplines depends predominantly on aerobic metabolism, including maximal and submaximal oxygen uptake, mitochondrial efficiency, substrate utilization, and cardiovascular function [2, 3]. However, competitive endurance events are not always performed at a constant, steady‐state intensity. Instead, they are characterized by dynamic fluctuations in pace, requiring athletes to repeatedly transition between steady‐state aerobic exercise and brief periods of high‐intensity effort, particularly during hills, surges, tactical positioning, transitions, and the final sprint to the finish line [4, 5].

From a physiological perspective, the determinants of endurance performance are collectively determined by the contribution of maximal oxygen uptake, lactate threshold, efficiency, and durability [2, 6]. These physiological determinants depend on the interaction of training‐induced adaptations of multiple organ systems, as endurance performance reflects the integrated function of energy metabolism, neuromuscular efficiency, acid–base balance, and fatigue resistance [7]. While oxidative phosphorylation predominates during prolonged exercise, anaerobic pathways, including phosphagen and glycolytic systems, remain critically involved during transient high‐intensity efforts [8]. The ability to tolerate and rapidly recover from these efforts may substantially influence race outcomes, especially in elite and subelite competitions, where marginal performance differences determine podium placement [9]. Consequently, nutritional strategies that support both sustained aerobic performance and high‐intensity exercise capacity have become increasingly relevant in endurance sports [10].

Two nutritional strategies have gained attention since the pioneering work of Professor Roger Harris in the fields of muscle metabolism: creatine and beta‐alanine supplementation [11, 12]. Creatine supplementation has traditionally been associated with improvements in strength, power, and short‐duration, high‐intensity exercise through increased intramuscular phosphocreatine availability and enhanced adenosine triphosphate (ATP) resynthesis [13]. Although endurance athletes were historically considered unlikely to benefit from creatine, emerging evidence suggests that the physiological effects of creatine may extend beyond purely anaerobic contexts [14]. Creatine supplementation has been linked to improvements in repeated sprint ability, neuromuscular function, training quality, glycogen storage, and postexercise recovery kinetics [15–17]. These adaptations may be particularly relevant in running, road cycling, and triathlon competitions, where the capacity to generate short bursts of high power output can influence race dynamics and finishing performance [4].

Beta‐alanine supplementation represents another ergogenic strategy with potential relevance to endurance athletes. As the rate‐limiting precursor for carnosine synthesis in skeletal muscle, beta‐alanine increases intramuscular carnosine concentrations, thereby enhancing intracellular buffering capacity [12]. This mechanism delays the accumulation of hydrogen ions during high rates of glycolysis, attenuating exercise‐induced acidosis, and fatigue [18]. While beta‐alanine has been extensively studied in high‐intensity exercise lasting one to ten minutes, endurance competitions frequently include segments where metabolic acidosis may become limiting, such as uphill running, cycling breakaways, accelerations, and end‐race sprints. In these contexts, enhanced buffering capacity may support performance by preserving muscle contractile function and delaying neuromuscular fatigue [19].

Importantly, the physiological demands of endurance sports suggest that no single metabolic pathway operates in isolation. Rather, successful performance relies on the coordinated interaction between aerobic metabolism, phosphagen availability, glycolytic flux, and fatigue modulation mechanisms. Within this framework, the combined use of creatine and beta‐alanine presents a compelling theoretical rationale. Despite these theoretical advantages, the scientific literature examining creatine and beta‐alanine individually or cosupplementation in endurance athletes remains scarce, with inconsistent findings and limited integrative discussion of underlying mechanisms. For the purposes of this review, the term *endurance athletes* encompasses runners, cyclists, triathletes, and ultraendurance athletes. Although these disciplines share a common reliance on aerobic metabolism, they differ substantially in biomechanical demands, exercise intensity distribution, event duration, power‐to‐weight requirements, and tactical characteristics, which may influence both physiological responses and the ergogenic effects of nutritional interventions. The evidence presented throughout this review should be interpreted within the specific context of each endurance discipline rather than generalized across all endurance sports [20].

Although several recent reviews have examined the effects of creatine or beta‐alanine supplementation in endurance exercise and others have focused on their efficacy across multiple exercise modalities [21–23], important knowledge gaps remain. Previous reviews have primarily evaluated these supplements independently, emphasized traditional aerobic performance outcomes, or synthesized evidence without considering the physiological and tactical characteristics that distinguish modern endurance competition. Therefore, the purpose of this narrative review is to synthesize and critically discuss the current evidence on creatine and beta‐alanine individual supplementation and cosupplementation in the context of endurance athletes aiming to optimize competitive performance and reach the podium. Given the scarcity of studies involving Tier 3–5 athletes (i.e., elite athlete classifications), we reviewed all clinical trials according to McKay et al. [20]. McKay and colleagues classify athletes as Tier 1 (recreationally active, participating in multiple sports), Tier 2 (trained/developmental, local‐level athlete training, 3 times per week with an intent to compete), Tier 3 (highly trained/national‐level athletes), Tier 4 (elite athletes competing at the international level), or Tier 5 (world‐class, Olympic and/or World Championship medalists). Emphasis is placed on physiological mechanisms, performance‐relevant outcomes, and the potential role of each supplement or cosupplementation as ergogenic aids.

## 2. Methods

A structured narrative review was conducted to critically examine the physiological rationale and practical applications of creatine supplementation, beta‐alanine supplementation, and their potential cosupplementation in endurance athletes. Literature searches were performed using the PubMed/MEDLINE, Scopus, and Web of Science databases for studies published from January 1, 1990, up to January 10, 2026. To establish the physiological basis for the mechanisms of action of creatine and beta‐alanine, seminal studies published before 1990 were also considered when relevant to phosphocreatine metabolism, skeletal muscle carnosine synthesis, and exercise bioenergetics. Search terms included combinations of keywords and Boolean operators related to endurance sports, supplementation, and exercise performance. Representative search terms included (“creatine” OR “creatine monohydrate”), (“beta‐alanine” OR “β‐alanine” OR “carnosine”), (“endurance athlete” OR “endurance exercise” OR “running” OR “marathon” OR “cycling” OR “cyclist” OR “triathlon” OR “triathlete”), combined with (“performance” OR “VO_2_max” OR “lactate threshold” OR “ventilatory threshold” OR “time trial” OR “time‐to‐exhaustion” OR “fatigue” OR “recovery” OR “sprint performance” OR “muscle glycogen” OR “buffering capacity”). Additional targeted searches were conducted to identify studies investigating physiological mechanisms potentially relevant to endurance performance, including phosphocreatine availability, intracellular buffering, muscle carnosine content, glycogen resynthesis, exercise‐induced inflammation, neuromuscular fatigue, and recovery.

Peer‐reviewed randomized controlled trials, crossover studies, observational studies, systematic reviews, meta‐analyses, consensus statements, and position stands published in English were considered eligible for inclusion. The study selection process was conducted in sequential stages. First, records identified through searches in PubMed, Scopus, and Web of Science were screened by title for relevance to the scope of the review. The abstracts of potentially eligible articles were subsequently evaluated, and studies considered relevant to the research question were retrieved for full‐text assessment. Studies investigating creatine and/or beta‐alanine supplementation in the context of endurance exercise and endurance‐related performance outcomes were analyzed. The reference lists of relevant reviews, consensus statements, and position stands were also screened to identify additional studies not retrieved through the initial database searches. Following the screening and selection process, a total of 19 articles were included and discussed in the structured narrative synthesis. Additional studies cited included systematic reviews and meta‐analyses, narrative reviews, seminal studies that provided foundational evidence within the field, and relevant consensus statements or position stands. These studies were included when considered relevant to contextualize the central research question, provide the physiological and theoretical background for creatine and beta‐alanine supplementation, and support the interpretation of the available evidence in endurance sports.

Study selection was conducted independently by multiple authors, and duplicated studies were removed. Studies were selected when they evaluated physiological responses, exercise performance outcomes, recovery variables, or mechanistic pathways associated with creatine and/or beta‐alanine supplementation. Particular emphasis was placed on outcomes relevant to endurance sports, including aerobic capacity, lactate and ventilatory thresholds, time‐trial performance, repeated high‐intensity efforts, sprint performance, fatigue resistance, recovery, muscle glycogen metabolism, body composition, and exercise‐induced muscle damage and inflammation. Non‐peer‐reviewed literature or studies lacking exercise‐related physiological or performance outcomes were excluded.

Because studies specifically involving highly trained endurance athletes remain limited, evidence from participants across different performance levels was considered. Particular attention was given to studies involving endurance disciplines such as running, cycling, triathlon, and ultraendurance events. When endurance‐specific evidence was unavailable, mechanistic studies involving healthy or physically active participants were included to provide physiological insights potentially relevant to endurance exercise. The evidence was synthesized narratively, emphasizing physiological plausibility, consistency of findings, supplementation protocols, participant characteristics, and practical applicability to endurance training and competition. Special consideration was given to race scenarios commonly observed in endurance sports, including repeated surges, climbing sections, transitions, and end‐race sprint efforts, as these race‐defining moments may represent the contexts in which creatine and beta‐alanine supplementation bioenergetically could exert the greatest ergogenic relevance.

## 3. Beta‐Alanine Supplementation for the Endurance Athlete

Beta‐alanine is a nonproteinogenic amino acid that serves as the rate‐limiting precursor for the synthesis of carnosine, an endogenous skeletal muscle buffer. Histidine, the other amino acid constituent of carnosine, is more abundant. Beta‐alanine can be obtained from the diet through the consumption of poultry and meat, but it is also produced endogenously in the liver. Although it is available in the diet and produced in the body, the amount necessary to act as an ergogenic aid depends on supplementation. Most of the studies register positive effects over a course of 4 weeks with a daily dose between 4 and 6 g [24]. Although carnosine was discovered around 1900 [25], only recently in the last 20 years has beta‐alanine drawn our attention [24]. A pioneer study conducted by Harris et al. [12] investigated the absorption of orally ingested beta‐alanine and its impact on skeletal muscle carnosine content in healthy individuals. Participants were supplemented with beta‐alanine at doses ranging from approximately 3.2 to 6.4 g/day over a period of 4 to 10 weeks, with muscle biopsies obtained from the vastus lateralis to assess intramuscular carnosine levels. The results demonstrated a marked and dose‐dependent increase in muscle carnosine concentration following supplementation, confirming beta‐alanine as the rate‐limiting precursor for carnosine synthesis in human skeletal muscle. These findings provide a strong mechanistic foundation for beta‐alanine supplementation as an ergogenic strategy, particularly in exercise conditions characterized by high glycolytic demand and increased reliance on intracellular buffering capacity.

In relation to physical exercise, Hill et al. [26] examined the effects of beta‐alanine supplementation on muscle carnosine content and high‐intensity exercise capacity in physically active men. Participants consumed 4.0–6.4 g/day of beta‐alanine for 10 weeks, and muscle biopsies from the gastrocnemius and soleus were used to quantify changes in intramuscular carnosine. Exercise capacity was assessed through cycling tests to exhaustion performed at intensities above VO_2_max. Beta‐alanine supplementation significantly increased muscle carnosine content by 58% after 4 weeks and 80% after 10 weeks. In parallel, total work done during cycling capacity tests improved by 13.0% after 4 weeks and 16.2% after 10 weeks, whereas no meaningful changes were observed in the placebo group. These findings indicate that beta‐alanine supplementation enhances high‐intensity exercise capacity, likely through increased intramuscular carnosine availability and improved intracellular buffering capacity.

The effects of beta‐alanine supplementation on endurance performance have been investigated using a range of laboratory and sport‐specific protocols, including time‐to‐exhaustion tests, incremental exercise, simulated road races, and time trials. Although findings remain inconsistent, studies have reported improvements in time‐to‐exhaustion [27, 28], 10‐km time‐trial performance [29], and physiological responses associated with exercise tolerance [28], whereas other investigations have observed no significant effects on endurance performance outcomes [30–34]. Specifically on endurance performance, a clinical trial investigated the effects of beta‐alanine supplementation combined with high‐intensity interval training (HIIT) on endurance performance and body composition in recreationally active men [35]. Participants supplemented with 6.0 g/day of beta‐alanine for 3 weeks, followed by 3.0 g/day for an additional 3 weeks, while completing a structured HIIT program. Performance outcomes included time‐to‐exhaustion, VO_2_peak, and time trial performance, alongside assessments of body composition. The beta‐alanine group showed a significantly greater improvement in time‐to‐exhaustion (+5.0%) compared with placebo, whereas VO_2_peak and time trial performance did not differ significantly between groups. In addition, beta‐alanine supplementation was associated with a greater increase in lean body mass (+1.1 kg) relative to placebo. These findings suggest that beta‐alanine may enhance training‐induced adaptations related to exercise tolerance and fatigue resistance, even when changes in classical aerobic markers such as VO_2_peak are not observed.

Glenn et al. [36] examined the effects of 28 days of beta‐alanine supplementation on high‐intensity cycling performance and blood lactate responses in masters women cyclists. Participants consumed 800 mg of beta‐alanine four times daily (3.2 g/day) for 28 days in a double‐blind, placebo‐controlled design. Performance was assessed using repeated high‐intensity cycling bouts and measures of blood lactate accumulation. Following supplementation, the beta‐alanine group demonstrated a significant improvement in time‐to‐exhaustion during high‐intensity cycling (+11.4%), whereas no meaningful change was observed in the placebo group. In addition, beta‐alanine supplementation was associated with a greater postexercise blood lactate response, suggesting an enhanced tolerance to high‐intensity metabolic stress. These findings indicate that short‐term beta‐alanine supplementation may improve high‐intensity exercise capacity in trained older women cyclists, likely through improvements in intracellular buffering capacity.

Milioni et al. [37] investigated whether beta‐alanine supplementation during HIIT intervention could enhance repeated sprint ability and attenuate neuromuscular fatigue. Recreationally active men supplemented with 6.4 g/day of beta‐alanine for 4 weeks while completing a HIIT protocol designed to stress anaerobic and neuromuscular systems. Performance outcomes included repeated sprint tests and markers of neuromuscular fatigue assessed before and after the intervention. Although both groups improved with training, beta‐alanine supplementation led to a significantly greater improvement in repeated sprint performance, particularly by reducing performance decrement across sprints, and was also associated with a lower degree of postexercise neuromuscular fatigue compared with placebo. These findings suggest that beta‐alanine may potentiate adaptations to HIIT by improving buffering capacity and fatigue resistance during repeated high‐intensity efforts.

Santana et al. [29] investigated the effects of beta‐alanine supplementation on 10‐km running performance in physically active adults. Participants consumed 6.4 g/day of beta‐alanine for 4 weeks in a double‐blind, placebo‐controlled design. Performance was assessed through a 10‐km running time trial conducted before and after the supplementation period. The beta‐alanine group demonstrated a significant improvement in running performance, with a reduction in total completion time of approximately 1.2% (∼1 min faster) compared with baseline, whereas no significant changes were observed in the placebo group. These findings indicate that beta‐alanine supplementation may enhance endurance performance in middle‐distance events, potentially by improving tolerance to sustained high‐intensity effort and delaying fatigue.

More recently, Marko et al. [28] investigated the effects of 4 weeks of beta‐alanine supplementation on aerobic performance in competitive middle‐ and long‐distance adolescent runners. Twenty‐seven runners completed maximal graded treadmill tests and submaximal running trials before and after supplementation, with the beta‐alanine group receiving 4.8 g/day (women) or 6.4 g/day (men). The main finding was a significant improvement in time‐to‐exhaustion in the beta‐alanine group, which increased by 6.5%, compared with only 1.4% in the placebo group, despite only trivial changes in VO_2_peak. In addition, during the submaximal trial, the beta‐alanine group showed reductions of approximately 4% in average heart rate and 4% in respiratory exchange ratio, suggesting improved physiological efficiency at a given workload. These findings reinforce that beta‐alanine may enhance endurance performance primarily by delaying fatigue and improving exercise tolerance rather than by increasing maximal aerobic capacity.

A systematic review and meta‐analysis was conducted to evaluate the effects of beta‐alanine supplementation on exercise performed within the aerobic–anaerobic transition zone, a domain highly relevant to endurance performance [38]. The review included studies investigating beta‐alanine protocols typically ranging from 3.2 to 6.4 g/day for 4 to 10 weeks, with outcomes focused on exercise capacity near lactate threshold, ventilatory threshold, and maximal aerobic speed. Overall, the meta‐analysis showed that beta‐alanine supplementation produced a small but significant positive effect on physical performance in this intensity domain, suggesting a meaningful ergogenic effect when exercise is performed near the upper limit of aerobic metabolism. The authors concluded that beta‐alanine may be particularly useful for improving performance in efforts requiring sustained intensity close to the aerobic–anaerobic transition, one more time likely due to enhanced intracellular buffering and delayed onset of fatigue.

Endurance exercise is a broad definition of exercise that includes activities with long duration that promotes metabolic adaptations in the muscles and elevates heart rate in a challenging way [39]. Among these metabolic adaptations, the ability to buffer the hydrogen ions produced by the active muscles in an exercise that is challenging is very important to sustain the exercise for longer periods or to be able to increase the intensity of the exercise even in shorter periods. Although the general idea of endurance exercise relates more to a continuous and moderate‐intensity effort, we can also consider endurance the notion of resisting a high‐intensity effort for a longer time. A recent meta‐analysis that investigated these two aspects of endurance in running economy published by Feng et al. [40] shows that for running economy, the HIIT, or high‐intensity interval efforts, are better than the moderate‐intensity continuous training (MICT). These results are based, among other elements, on the postponement of lactate plus hydrogen ions produced by exercising muscles, exactly the metabolic process where beta‐alanine can be an effective ergogenic agent.

Tempo run, a specific type of running training, aims to sustain a fast pace for a longer period. This training mode, which is actually very popular, has an intensity and duration combination right between the moderate‐intensity continuous training and the high‐intensity intervals [41]. These running examples are only a particularity of the authors that can easily be translated to other types of sports or exercises where the intensity and duration function is considered and needs improvement. This is the rationale that led us to the position that beta‐alanine can be an effective ergogenic aid to endurance exercise. Regarding the possibility of beta‐alanine being a relevant ergogenic aid during final sprint efforts, Van Thienen et al. [42] evaluated the effects of beta‐alanine supplementation on sprint performance in trained endurance cyclists. Participants consumed 4 g/day of beta‐alanine for 8 weeks in a placebo‐controlled design and completed a simulated cycling time trial that incorporated a final sprint effort. While beta‐alanine supplementation did not significantly improve overall time trial performance, it significantly enhanced final sprint power output, with cyclists producing 11.4% more power during the finishing sprint compared with placebo. In addition, beta‐alanine supplementation attenuated the decline in performance during the final high‐intensity phase of the test. These findings suggest that although beta‐alanine may not substantially alter steady‐state endurance performance, it may provide a meaningful ergogenic benefit during decisive high‐intensity moments in endurance events, such as end‐race sprinting.

From a clinical perspective, we provided a narrative review examining whether the skeletal muscle carnosine response to beta‐alanine supplementation can be optimized through adjustments in dosing strategies and supplementation protocols [43]. We synthesized evidence from human studies investigating factors such as daily dose, duration of supplementation, dosing frequency, coingestion with meals, and interindividual variability in responsiveness. Overall, our review highlighted that beta‐alanine supplementation consistently increases muscle carnosine content, with typical doses of 3.2–6.4 g/day over 4–12 weeks producing substantial elevations, although the magnitude of response varies considerably between individuals. There was also evidence suggesting that higher cumulative doses, longer supplementation periods, and possibly coingestion with meals may further enhance carnosine loading. Collectively, the review concluded that beta‐alanine is an effective strategy for increasing muscle carnosine stores and that optimizing supplementation protocols may maximize its ergogenic potential, particularly for exercise modes involving high‐intensity efforts and metabolic acidosis. All these studies and their results, at this moment in time, give us the notion that beta‐alanine supplementation, on a 4 to 6 g per day dosage for around 28 days, can be used as an effective ergogenic aid for endurance sports, such as long runs or long bike rides with high, but not maximum, intensity, working as a buffering agent delaying fatigue in high‐intensity efforts (Table 1). Moreover, it could be an interesting strategy to provide more durability/resilience to athletes, as the ability to resist functional decline following acute and/or chronic stressors during races is also determinant in endurance performance.

**TABLE 1:** Summary of studies investigating beta‐alanine supplementation on endurance performance.

Study	Participants	Supplementation protocol	Endurance protocol	Main findings
Bellinger and Minahan [32]	14 trained men cyclists	BA: 6.4 g/day for 4 weeks followed by 1.2 g/day for 5 weeks or Pla (dextrose monohydrate)	4‐km and 10‐km cycling time trials	Beta‐alanine did not improve 4‐km or 10‐km time‐trial performance (4‐km TT ↔; 10‐km TT ↔).
Chung et al. [31]	27 endurance‐trained cyclists and triathletes	BA: 6.4 g/day for 6 weeks or Pla (maltodextrin)	Incremental cycling test to exhaustion	No improvements in TTE, blood lactate, RPE or HR (TTE ↔).
Furst et al. [33]	12 healthy middle‐aged adults	BA: 2.4 g/day for 28 days or Pla (microcrystalline cellulose)	Cycling TTE test at 70% VO_2_peak	No improvements in TTE or blood lactate (TTE ↔).
Ghiasvand et al. [27]	39 healthy men college students	BA: 2 g/day for 6 weeks or Pla (dextrose)	Incremental cycling test to exhaustion	TTE ↑ and postexercise blood lactate ↓; VO_2_max ↔.
Marko et al. [28]	23 competitive adolescent middle‐ and long‐distance runners	BA: 6.4 g/day (men) or 4.8 g/day (women) for 4 weeks or Pla (maltodextrin)	Maximal graded treadmill test and 1500‐m submaximal treadmill test	TTE ↑6.5%; VO_2_peak ↔; VT2 ↑; HR and RER ↓ during submaximal running.
Perim et al. [34]	17 competitive cyclists	BA: 6.4 g/day for 28 days or Pla	Simulated road race with repeated 10‐s sprints followed by a 4‐km uphill TT	Muscle carnosine ↑; repeated sprint and 4‐km TT ↔.
Santana et al. [29]	16 physically active men	BA: 5 g/day for 23 days or Pla (resistant starch)	10‐km treadmill time trial	10‐km TT ↑; postexercise blood lactate ↓.
Smith‐Ryan et al. [30]	50 healthy men and women	BA: 4.8 g/day for 28 days or Pla (maltodextrin)	Running TTE test at 90% *V*_max_	No improvements in TTE or blood lactate in men or women (TTE ↔).

*Note:* ↑, improvement; ↓, reduction; ↔, no significant change. Pla, placebo; VO_2_max, maximal oxygen uptake; VO_2_peak, peak oxygen uptake; VT2, second ventilatory threshold.

Abbreviations: BA, beta‐alanine; HR, heart rate; RER, respiratory exchange ratio; RPE, rating of perceived exertion; TT, time trial; TTE, time‐to‐exhaustion.

It is important to mention that beta‐alanine supplementation should be viewed as one component of an integrated nutritional strategy rather than an isolated intervention. Given its proposed role in increasing intracellular buffering capacity, beta‐alanine may complement ergogenic aids targeting different physiological mechanisms (Figure 1), including nitrate, caffeine, and sodium bicarbonate, although evidence supporting additive or synergistic effects remains limited and inconsistent [24, 44].

**FIGURE 1:**
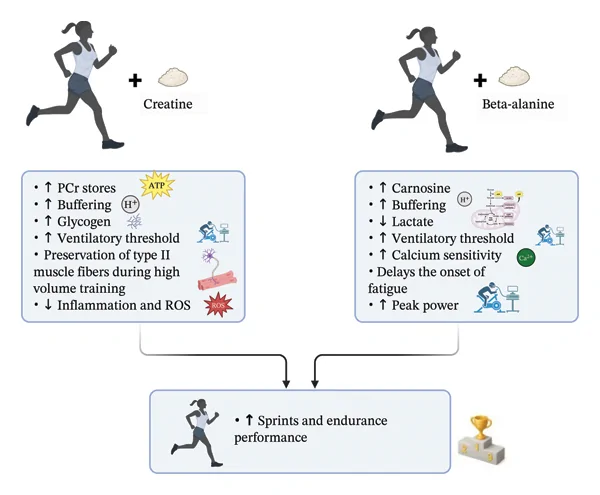
Possible mechanisms of action of creatine and/or beta‐alanine supplementation in endurance athletes.

### 3.1. Potential Practical Applications

Based on the current evidence mentioned and discussed throughout this review article and the authors’ practical interpretation, the following practical applications are provided:

- Current evidence supports chronic supplementation with approximately 3.2–6.4 g/day (or ∼0.05–0.06 g/kg/day) for at least 4 weeks to substantially increase skeletal muscle carnosine concentrations, with longer supplementation periods producing further increases in muscle carnosine content. To minimize paraesthesia, the total daily dose should be divided into smaller doses (approximately 0.8–1.6 g per serving) distributed throughout the day. Slow‐release formulations may further improve tolerability.
- Beta‐alanine appears to be most beneficial during exercise lasting approximately 1–10 min or during prolonged endurance events that incorporate repeated efforts performed near or above the aerobic–anaerobic transition zone, where intracellular acidosis is more likely to contribute to fatigue.
- The ergogenic effects of beta‐alanine appear to be modest, with reported performance improvements of approximately 2%–3% in recreationally active individuals and 0.5%–1.0% in elite athletes, emphasizing that supplementation should complement, rather than replace, evidence‐based training and nutritional strategies.

## 4. Creatine Supplementation for the Endurance Athlete

Phosphocreatine (PCr) acts as a bridge between energy production (mitochondria) and energy consumption (myofibrils). It transports high‐energy phosphates from the mitochondria (where they are created) to the contractile machinery of the muscle. Despite the recent popularity of creatine supplementation, it is not a new supplement. The first study conducted was in 1926 in which it was supplemented with 10–20 g/day for 29–44 days to analyze the fate of creatine when administered to men [45]. The two authors served as the subjects for the present experiments, subsisting upon a creatine‐free diet for 6 and 8 weeks respectively, and they found that creatine can be retained in the organism. Another landmark study in 1992 conducted by Professor Roger Harris first demonstrated in humans that multiple daily supplementation with creatine 20–30 g/day (5 g per dose) could increase total intramuscular creatine (TCr = PCr + Cr) levels by up to 20% [11]. Subsequent studies have also demonstrated the effectiveness of creatine supplementation in increasing muscle creatine levels, including with more gradual exercise regimens [46] and it is not dependent on the timing of supplementation [47]. However, the effects of creatine supplementation and endurance performance still needs more research.

Endurance performance is influenced by many factors. Elevation changes, race dynamics, course layout, and the athlete’s goals and racing strategy are some of the factors that can affect an athlete’s pace [48]. As a result, endurance races are rarely completed at a steady pace but often include multiple intermediate stages performed at maximal or supramaximal intensities, such as finishing in sprints or overtaking other athletes [48]. However, considering that endurance races typically have a predominance of aerobic metabolism, it is generally believed that creatine has no effect on endurance performance or oxidative capacity (i.e., VO_2_max) and may even be detrimental [49]. The perceived decrease in endurance performance may be related to the challenge of short‐term creatine to intracellular and/or endogenous water retention, leading to weight gain, which is thought to impair endurance performance during exercise (e.g., running) [50]. In weight‐bearing endurance sports such as marathon running and trail running, it is hypothesized that small increases in body mass due to creatine supplementation could influence running economy, metabolic cost, and uphill performance.

A systematic review and meta‐analysis evaluated 143 randomized controlled trials on the effects of creatine supplementation on body composition [51]. Creatine led to small but significant increases in body mass (+0.86 kg) and fat‐free mass (+0.82 kg), along with a modest reduction in body fat percentage (−0.28%). A clinical trial evaluated in habitually active to well‐trained male subjects reported that creatine supplementation (20 g/day for 6 days) impaired 6‐km running performance, likely due to an increase in body mass (+0.9 kg) [52]. This contrasts with findings in cycling, suggesting that body mass gains from creatine may negatively affect weight‐bearing activities such as running more than non‐weight‐bearing ones such as cycling [53]. Another systematic review and meta‐analysis included 13 studies investigating creatine monohydrate supplementation in endurance‐trained individuals [54]. The pooled results showed no significant effect on endurance performance. The authors associated the lack of improvement with increased body mass from supplementation, particularly in running. However, creatine may benefit aerobic performance in sports where added mass does not elevate energy cost and muscular strength plays a key role. More clinical trials in runners and trail runners are required to analyze this hypothesis and should be interpreted alongside the potential physiological benefits of creatine supplementation in some specific contexts.

In cycling trials, the magnitude of change observed in most investigations is considerably small, typically ranging from 0.5 to 1.0 kg, and appears to occur predominantly during the early phase of supplementation [55]. Supporting this view, evaluations of body composition in Tier 2–3 men cyclists using dual‐energy x‐ray absorptiometry after a short‐term creatine loading protocol (20 g/day for 5 days), followed by a brief maintenance phase (3 g/day for 2–9 days), did not reveal significant alterations in either total or lean body mass, with changes of approximately 1.2% relative to baseline [56]. Such evidence indicates that concerns regarding substantial creatine‐induced changes in body mass or composition may be exaggerated, particularly in trained endurance athletes. Conversely, creatine supplementation can increase intramuscular PCr, improve buffering capacity, enhance glycogen resynthesis and muscle glycogen content mainly when combined to carbohydrate, and reduce oxidative stress and inflammation [57]. As explained in detail in a narrative review conducted by Forbes et al. [21], these purported benefits may offset weight gain and have a positive impact on endurance performance and recovery.

One proposed mechanism through which creatine supplementation may benefit endurance athletes is through enhanced muscle glycogen, reducing the phosphofructokinase activity and rising the glycogen resynthesis and storage, also by upregulation and increase in GLUT‐4 levels and insulin receptor sensitivity with concomitant changes in cell volume [58]. An important study in the field analyzed 14 healthy men who cycled to exhaustion at 70% peak oxygen uptake (VO_2_) and subsequently consumed a high‐carbohydrate diet (> 80% of calories as carbohydrates) with and without 20‐g/day creatine supplementation [17]. Creatine supplementation increased intramuscular creatine stores and muscle glycogen content within 24 h compared to the placebo group, and these elevated levels persisted for 6 days. Another study involved 43 recreationally active men to verify the effects of four weeks of creatine supplementation and high‐intensity interval training on cardiorespiratory fitness [59]. Participants were then randomly assigned to a 10‐g/day creatine supplementation group, a placebo group, or a nontraining control group. Only the creatine group demonstrated a significant increase in ventilatory threshold (16% vs. 10% in the placebo group). These findings suggest that creatine supplementation may enhance specific physiological adaptations, particularly improvements in ventilatory efficiency, which is interesting for endurance performance.

Creatine supplementation can also preserve fast‐twitch fibers during high‐volume training in animal models [60]. This study examined whether creatine loading could prevent fast‐to‐slow muscle fiber transitions induced by chronic low‐frequency electrical stimulation (CLFS) in rat fast‐twitch muscles. Rats received either creatine plus dextrose or dextrose alone for 10 days before and during 10 days of CLFS. Creatine loading significantly increased total creatine, PCr, and intracellular phosphorylation potential (IPP). In Cr‐CLFS muscles, expression of slow‐type myosin heavy chain (MyHC‐I and MyHC‐IIa) was suppressed compared to dextrose‐CLFS, indicating maintenance of the fast phenotype. Both Cr‐CLFS and dextrose‐CLFS showed increased oxidative enzyme activity, but only dextrose‐CLFS had reduced glycolytic capacity. Creatine also preserved glycolytic potential and increased SERCA2 and parvalbumin expression, suggesting enhanced energy buffering and calcium handling. These findings indicate that creatine supplementation can maintain a fast‐twitch glycolytic phenotype and also adapt to training stimulus by increasing oxidative capacity in animal models. However, we must emphasize that results from animal model studies should not be extrapolated to humans, and we recommend that studies in humans be conducted to verify these outcomes.

Analyzing a possible action as an intracellular buffer, since PCr hydrolysis consumes a hydrogen ion, Oliver et al. [61] demonstrated in recreationally active men that creatine supplementation (20 g/day for 6 days) was shown to reduce blood lactate concentrations during incremental cycling exercise at the end of each stage of the test, indicating a significant condition effect (*p* = 0.041), alongside a nonsignificant increase in power at lactate threshold (+15 W) and a trend toward improved time‐to‐exhaustion (+0.7 min). These findings suggest that creatine may attenuate metabolic acidosis during exercise, potentially via enhanced PCr availability and intracellular buffering capacity, thereby contributing to delayed fatigue during high‐intensity efforts embedded within endurance exercise. We emphasize that this is a unique article and that this potential to act as a buffering agent requires further in‐depth clarification.

Another potential application of creatine supplementation to the endurance athlete is reducing inflammatory markers and enhancing postexercise recovery [21]. A study investigated the effects of creatine supplementation on markers of muscle damage and inflammation following a 30‐km race in trained marathon runners [62]. Participants received either creatine (4 × 5 g/day) plus maltodextrin or maltodextrin alone for 5 days prior to the race. Blood samples were collected pre‐ and 24 h postrace. In the control group, postrace levels of creatine kinase (CK), lactate dehydrogenase (LDH), prostaglandin E2 (PGE2), and tumor necrosis factor‐alpha (TNF‐α) increased significantly, indicating muscle damage and inflammation. Creatine supplementation attenuated the rise in CK (−19%), PGE2 (−60.9%), and TNF‐α (−33.7%) and completely prevented the elevation in LDH. These findings suggest that creatine may reduce exercise‐induced muscle damage and systemic inflammation in endurance athletes. In a systematic review and meta‐analysis, creatine supplementation during graded exercise tests enhanced ventilatory threshold, had no effect on relative VO_2_max, time‐to‐exhaustion, or maximal power output but decreased absolute VO_2_max compared to placebo [49]. Importantly, this particular review has considerable limitations, such as including both healthy and unhealthy participants, only three studies estimated VO_2_max, four of 19 studies were sedentary participants and did not include any exercise intervention, and one study was conducted using a resistance training protocol. Despite these limitations, in a subanalysis they found that creatine supplementation did not alter maximal power output or time‐to‐exhaustion but did enhance ventilatory threshold.

The effects of creatine supplementation on endurance performance have been investigated for more than 3 decades using both time‐trial and time‐to‐exhaustion protocols. While improvements in traditional endurance outcomes remain inconsistent, several studies have demonstrated beneficial effects on time‐to‐exhaustion [63–65] and high‐intensity performance during or following prolonged endurance exercise [66, 67]. To analyze the effects of creatine supplementation on endurance in a more realistic race approach, regarding cycling time trial performance, a study was performed not only at a steady pace, but also considering sprints [53]. The authors investigated the effects of creatine supplementation (20 g/day for 5 days, then 3 g/day for 9 days) combined with either high (12 g/kg) or moderate (6 g/kg) carbohydrate intake in 18 elite cyclists. Athletes completed a 120‐km time trial with alternating 1‐km and 4‐km sprints every 10 km. Creatine supplementation significantly improved power output in late‐stage sprints and also showed considerable effect during a hill climbing stage. A post‐trial treadmill test to exhaustion (90% VO_2_peak at 8% incline) showed that creatine‐induced body mass gain did not impair performance. These results suggest that creatine plus carbohydrate may enhance sprint capacity without negatively affecting endurance performance in elite cyclists.

Considering that a carbohydrate loading protocol was performed, the authors did not detect a significant difference in overall performance as a result of either creatine or carbohydrate loading; an increase in pretime trial weight did not affect the weight‐sensitive hill climbing at the end of the trial, which suggests that avoiding creatine supplementation in endurance cycling due to weight gain and/or slower result times may be unjustified as the augment in body mass can be attributed mainly to the carbohydrate loading. Another study investigated the effects of creatine supplementation (6 g/day for 5 days) on interval performance during an endurance protocol in regionally competitive triathletes [68]. Athletes cycled at 3 mmol·L^−1^ lactate threshold for 30 min, followed by 10 × 15‐s high‐intensity intervals (7.5 W/kg), short recovery periods, and a second round of intervals with another 30‐min effort. Creatine supplementation increased interval power output by 18% and extended time‐to‐exhaustion by 4 min in participants who previously failed to complete the final 30‐min bout. These findings highlight creatine’s potential to enhance high‐intensity efforts within endurance events.

According to a recently published consensus statement commissioned by the Union Cycliste Internationale (UCI), creatine supplementation has the capacity to improve sprint performance during endurance cycling events and/or events in which intermediate or final sprints determine how the race will be contested, as it seems to offer benefits to the performance of exercise that involves rapid generation of high power outputs, which are often key race‐defining moments for several cycling disciplines [69]. Considering these available data regarding creatine supplementation and potential mechanisms of how creatine supplementation can enhance endurance performance (Figure 1), the current literature indicates that some endurance athletes can consider creatine supplementation as an adjuvant strategy (Table 2).

**TABLE 2:** Summary of studies investigating creatine supplementation on endurance performance.

Study	Participants	Supplementation protocol	Endurance protocol	Main findings
Balsom et al. [52]	18 well‐trained men	20 g/day × 6 d or Pla	6‐km TT + TTE at 120% vVO_2_max	Impaired 6‐km TT; TTE unchanged.
Engelhardt et al. [68]	12 competitive triathletes	6 g/day × 5 d or Pla	30‐min cycling + repeated 15‐s sprints	Interval work ↑18%; aerobic performance unchanged; TTE ↑∼4 min in nonfinishers.
Jacobs et al. [63]	26 recreational/competitive athletes	20 g/day × 5 d or Pla	Cycling TTE at 125% VO_2_max	TTE ↑∼9%.
Rico‐Sanz et al. [64]	14 trained cyclists	20 g/day × 5 d or Pla	Alternating exercise at 30/90% MPO until exhaustion	TTE ↑.
Schaffer et al. [65]	11 recreationally active men	20 g/day × 5 d + 2 g/day or Pla	TTF above critical power	TTF ↑∼11%.
Tomcik et al. [53]	18 well‐trained cyclists	20 g/day × 5 d + 3 g/day × 9 d or Pla	120‐km TT + sprints + incline TTE	Final sprints ↑; overall TT and incline TTE unchanged.
Vandebuerie et al. [66]	12 elite cyclists	25 g/day × 4 d or Pla	2.5‐h cycling + TTE + five 10‐s sprints	Sprint performance ↑8%–9%; TTE unchanged.
Van Loon et al. [67]	20 healthy young men	20 g/day × 5 d + 2 g/day × 37 d or Pla	20‐min TT + twelve 12‐s sprints	Sprint performance ↑; 20‐min TT unchanged.

*Note:* ↑, improvement; ↔, no significant change; Pla, placebo; VO_2_max, maximal oxygen uptake; vVO_2_max, velocity associated with maximal oxygen uptake.

Abbreviations: MPO, maximal power output; TT, time trial; TTE, time‐to‐exhaustion; TTF, time‐to‐fatigue.

Finally, as discussed in this section, creatine can assist in preserving fast‐twitch muscle fibers during high‐volume training and creatine combined with carbohydrates can increase muscle glycogen. In regard to gaining body mass, creatine can induce a small gain, but an individualized approach may be warranted for weight‐bearing sports. Other areas of research should be investigated in this field, such as creatine supplementation in the off‐season of the endurance athlete, during a high‐volume training phase, its effects on inflammation and muscle damage mainly on ultraendurance athletes, and on the immunosuppression that affects this athlete. Creatine supplementation should be considered within the broader context of evidence‐based sports nutrition rather than as a stand‐alone ergogenic strategy. For endurance athletes, nutritional interventions with stronger evidence for improving endurance performance, such as carbohydrate availability, carbohydrate ingestion during exercise, hydration strategies, and caffeine supplementation, should generally be prioritized according to the athlete’s event characteristics and performance goals [70, 71].

### 4.1. Potential Practical Applications

Based on the current evidence mentioned and discussed throughout this review article and the authors’ practical interpretation, the following practical applications are provided:

- Creatine monohydrate remains the preferred formulation.
- A traditional loading protocol (20 g/day or ∼0.3 g/kg/day, divided into four daily doses for 5–7 days), followed by a maintenance dose of 3–5 g/day (or ∼0.03 g/kg/day), appears effective for rapidly increasing intramuscular creatine stores. Alternatively, daily supplementation with 3–5 g/day for approximately 4 weeks may achieve similar elevations in muscle creatine content while minimizing the transient increase in body mass associated with water retention.
- Creatine supplementation may be particularly relevant during training phases or races characterized by frequent high‐intensity interval sessions, repeated surges, hill repetitions, and race‐specific efforts, where phosphocreatine turnover and glycolytic energy production are substantially increased.
- For athletes competing in weight‐bearing endurance sports, particularly events with substantial climbing or prolonged running, supplementation strategies should be individualized, balancing the potential performance benefits against the possibility of transient body mass increases.
- Because individual responsiveness varies, athletes should trial creatine during training rather than immediately before an important competition, allowing adequate time to evaluate tolerance and any potential effects on body mass or performance.

## 5. Creatine and Beta‐Alanine Co‐Supplementation for the Endurance Athlete: A Synergistic Effect?

The influence of creatine and beta‐alanine cosupplementation on aerobic capacity and endurance performance has been examined in a limited number of controlled studies [70]. A clinical trial evaluated the effects of 4 weeks of beta‐alanine and creatine supplementation on several markers of endurance performance [72]. In a randomized, double‐blind, placebo‐controlled design, 55 men were allocated to one of four groups: placebo, creatine, beta‐alanine, or combined beta‐alanine and creatine. Before and after the supplementation period, participants completed a graded cycling test to assess VO_2_peak, time‐to‐exhaustion, and power output, as well as VO_2_ and relative VO_2_peak values corresponding to ventilatory and lactate thresholds. No significant differences were detected between groups for any endurance‐related outcomes. Nevertheless, a significant time effect was observed within the combined supplementation group, which demonstrated improvements in five of the eight variables assessed. Collectively, these findings indicate that although improvements from baseline were observed within the creatine and beta‐alanine cosupplementation group, these changes were not significantly different between groups. Therefore, these findings do not provide clear evidence of an additional ergogenic effect of cosupplementation.

In a study involving college‐aged recreationally active women, 4 weeks of supplementation combined with structured endurance and cycling training resulted in no significant differences in the VO_2_peak between placebo, creatine, beta‐alanine, or combined supplementation groups [73]. Thirty‐two women participated in this randomized, double‐blind, placebo‐controlled trial and were assigned to one of four supplementation conditions: beta‐alanine alone, creatine alone, combined beta‐alanine and creatine, or placebo. Participants completed testing sessions at baseline, after 7 days, and at the conclusion of the supplementation period (Day 28). Each testing visit included a graded cycling exercise test to determine the VO_2_peak and lactate threshold, as well as a series of Wingate tests to evaluate anaerobic performance. Neither beta‐alanine alone nor cosupplementation improved maximal aerobic capacity, while creatine supplementation showed a small, nonsignificant tendency toward increased time‐to‐exhaustion at VO_2_peak.

Beyond maximal aerobic capacity, some investigations have examined whether cosupplementation influences fatigue‐related thresholds during incremental exercise. Stout et al. [74] evaluated whether 28 days of supplementation with beta‐alanine and creatine monohydrate influences the development of neuromuscular fatigue in untrained men. Fifty‐one men volunteers were enrolled in a randomized, double‐blind, placebo‐controlled design and allocated to one of four supplementation protocols: placebo, creatine monohydrate, beta‐alanine, or a combined beta‐alanine and creatine regimen. Before and after supplementation, participants completed a continuous incremental cycling test. Postintervention analyses, adjusted for baseline values, revealed higher physical working capacity at neuromuscular fatigue threshold in both the beta‐alanine and combined supplementation groups compared with placebo. In contrast, no differences were observed between creatine and placebo, nor between the combined and beta‐alanine‐only conditions. Collectively, these findings suggest that beta‐alanine supplementation can delay the onset of neuromuscular fatigue, whereas creatine monohydrate does not provide additional or synergistic benefits for this outcome.

Recently, Ashtary‐Larky et al. [23] conducted a systematic review examining the effects of creatine and beta‐alanine cosupplementation on exercise performance and body composition. Eligible studies included adults who received combined creatine and beta‐alanine supplementation for at least 4 weeks and compared outcomes with creatine or beta‐alanine alone, assessing exercise performance and/or body composition. Overall, cosupplementation did not provide additional improvements in aerobic endurance capacity, including VO_2_max, lactate threshold, or time‐to‐exhaustion. Collectively, these findings indicate that creatine and beta‐alanine cosupplementation does not provide additional advantages for aerobic capacity compared with single‐supplement strategies. Collectively, these findings indicate that cosupplementation of beta‐alanine and creatine does not appear to meaningfully provide a synergistic effect of enhanced aerobic performance or thresholds in the context of endurance exercise. Taking into account the limited number of controlled studies published that evaluated the cosupplementation, most available evidence indicates a negligible effect on maximal aerobic capacity. Thus, across both men and women cohorts, their combination does not appear to meaningfully enhance VO_2_max or VO_2_peak beyond the physiological adaptations induced by endurance training alone or by creatine or beta‐alanine isolated effects [21, 22].

Important to mention, these three studies have considerable limitations, which makes it challenging to compare each study. Several methodological and population‐related limitations complicate direct comparison and interpretation of their findings regarding creatine and beta‐alanine supplementation. First, the participant profiles differed substantially across studies, including untrained men [74], men without specification of the training level [72], and recreationally active women [73], which limits external validity for competitive endurance athletes and introduces sex‐ and training‐status–specific physiological variability in supplementation responsiveness. Second, the primary outcomes and testing methodologies were heterogeneous. Zoeller et al. [72] and Kresta et al. [73] emphasized aerobic performance metrics such as VO_2_peak, ventilatory threshold, lactate threshold, and time‐to‐exhaustion. Stout et al. [74] focused on neuromuscular fatigue using an electromyography‐derived fatigue threshold, a measure more closely related to high‐intensity tolerance than classical endurance performance. Differences in supplementation protocols further difficult interpretation, including variation in dosing strategies, use of creatine loading phases, and total daily beta‐alanine intake, as well as relatively short intervention durations (≈ 4 weeks), which may be insufficient to elicit maximal physiological adaptations, particularly for endurance‐related outcomes. Finally, most studies involved concurrent training or repeated testing, raising the possibility that training adaptations and test familiarization masked supplementation‐specific effects. Overall, the available evidence investigating the synergistic effects of creatine and beta‐alanine supplementation in endurance sports remains limited. The interaction between phosphagen availability and buffering capacity has not yet been confirmed in competitive endurance athletes. Consequently, the findings from existing studies should be interpreted with caution, as they may not adequately reflect the potential effects of cosupplementation in well‐trained endurance athletes (Table 3).

**TABLE 3:** Summary of studies investigating creatine and beta‐alanine cosupplementation on endurance performance.

Study	Participants	Supplementation protocol	Endurance protocol	Main findings
Kresta et al. [73]	32 recreationally active women	BA: 0.1 g·kg^−1^ day^−1^ + Cr: 0.3 g·kg^−1^ day^−1^ for 1 week followed by 0.1 g·kg^−1^ day^−1^ for 3 weeks or Pla	Incremental cycling test to determine VO_2_peak, ventilatory threshold, and lactate threshold	Cosupplementation did not improve VO_2_peak, ventilatory threshold, lactate threshold, or time‐to‐exhaustion compared with placebo or either supplement alone (VO_2_peak ↔; VT ↔; LT ↔; TTE ↔).
Stout et al. [74]	51 untrained healthy men	BA: 6.4 g/day for 6 days, then 3.2 g/day for 22 days + Cr: 21 g/day for 6 days, then 10.5 g/day for 22 days or Pla (dextrose)	Physical working capacity at the fatigue threshold (PWCFT) during incremental cycle ergometry	Cosupplementation improved physical working capacity at the fatigue threshold compared with placebo but not beyond beta‐alanine alone (PWCFT ↑).
Zoeller et al. [72]	55 untrained healthy men	BA: 6.4 g/day for 6 days, then 3.2 g/day for 22 days + Cr: 21 g/day for 6 days, then 10.5 g/day for 22 days or Pla (dextrose)	Incremental cycling test to determine VO_2_peak, ventilatory threshold, lactate threshold, and time‐to‐exhaustion	Cosupplementation improved several within‐group physiological variables, but no significant between‐group differences were observed for VO_2_peak, ventilatory threshold, lactate threshold, or time‐to‐exhaustion (VO_2_peak ↔; VT ↔; LT ↔; TTE ↔).

*Note:* Cr, creatine monohydrate; Pla, placebo; PWCFT, physical working capacity at the fatigue threshold; VO_2_peak, peak oxygen uptake; ↑, improvement; ↔, no significant change.

Abbreviations: BA, beta‐alanine; LT, lactate threshold; TTE, time‐to‐exhaustion; VT, ventilatory threshold.

## 6. Discussion and Conclusion

Taken together, the available literature suggests that beta‐alanine and creatine supplementation should not be interpreted as universal ergogenic strategies for all endurance athletes, but rather as context‐dependent tools whose usefulness is likely influenced by the athlete’s event demands, training structure, competitive level, and performance objective. The current body of evidence supports beta‐alanine as a potentially useful supplement for endurance scenarios that involve sustained exercise near the aerobic–anaerobic transition zone, repeated surges, uphill efforts, and end‐race sprinting, whereas creatine appears to be more relevant in race situations requiring repeated high‐intensity bursts, sprint capacity, glycogen resynthesis, and recovery between efforts. However, when translated into real‐world endurance practice, the expected value of these supplements likely depends more on whether the athlete’s training and competition profile actually exposes them to the types of demands these supplements are most likely to influence.

From a mechanistic perspective, the ergogenic potential of creatine and beta‐alanine supplementation in endurance athletes is likely greatest when training or competition imposes substantial demands on both the phosphagen and glycolytic energy systems. Although endurance performance is predominantly supported by oxidative metabolism, many race‐defining moments, including repeated surges, attacks, steep climbs, transitions, breakaways, and finishing sprints, require rapid ATP resynthesis through phosphocreatine degradation and accelerated glycolytic flux. Under these conditions, creatine may support performance by increasing phosphocreatine availability, enhancing ATP buffering and resynthesis, whereas beta‐alanine may attenuate exercise‐induced acidosis through increased intramuscular carnosine concentrations and intracellular buffering capacity. Future studies should evaluate creatine and beta‐alanine supplementation using exercise protocols that place greater demands on the phosphagen and glycolytic energy system.

One important factor to consider before translating these findings into clinical practice is training periodization. The rationale for prescribing creatine and/or beta‐alanine may be stronger during phases of training that include a greater density of high‐intensity interval sessions, repeated climbs, race‐pace intervals with surges, threshold work, finishing kick sessions, or competition‐specific blocks. In contrast, during phases dominated by high‐volume, low‐intensity aerobic work, the practical ergogenic relevance of these supplements may be less evident. Thus, supplementation should likely be periodized in parallel with the training periodization, rather than prescribed in a static, year‐round manner. For example, beta‐alanine may be particularly useful during periods emphasizing tolerance to sustained high‐intensity efforts and metabolic acidosis, whereas creatine may be strategically valuable during blocks that require repeated sprint work, high‐intensity overload, or enhanced glycogen support during dense training microcycles.

From the perspective of exercise intensity domains, the potential ergogenic effects of creatine and beta‐alanine are likely to become more relevant as exercise intensity approaches and exceeds the aerobic–anaerobic transition. The exercise within the heavy domain (approximately Zone 3 and the lower portion of Zone 4), particularly around the lactate threshold, progressively increases the contribution of anaerobic glycolysis and hydrogen ion accumulation. As intensity further increases into the severe domain (upper Zone 4 and Zone 5), repeated reliance on phosphocreatine resynthesis, glycolytic ATP production, and intracellular buffering becomes substantially greater. Consequently, creatine and beta‐alanine supplementation may theoretically provide their greatest ergogenic benefit during training sessions and competitive situations involving prolonged efforts near or above the aerobic–anaerobic transition. Nevertheless, this interpretation is mainly supported by physiological rationale and laboratory‐based evidence, highlighting the need for endurance‐specific studies evaluating supplementation responses across different exercise intensity domains.

Another central issue is the athlete’s competitive goal. The potential benefits of creatine and beta‐alanine are likely to differ for an athlete aiming to reach the podium than for one whose main objective is to finish the race. In highly competitive contexts, where race outcomes are often determined by tactical surges, sprint finishes, or the ability to maintain pace under accumulated fatigue, even small improvements in repeated high‐intensity tolerance, buffering capacity, or finishing ability may be meaningful. In contrast, for recreational or novice athletes focused on completion, enjoyment, or pacing consistency, the magnitude of benefit may be substantially smaller and perhaps not worth prioritizing over more established nutritional interventions such as carbohydrate availability, hydration, and training the gut. Therefore, expected effect size should always be interpreted relative to the athlete’s performance objective, not merely statistical significance. Accordingly, the athlete’s experience and performance tier should also be central to supplementation decisions.

A major limitation of the current literature is that most supplementation studies have been conducted in Tier 1–2 participants, including recreationally active, untrained, or moderately trained individuals, whereas there is a clear lack of studies in Tier 3–5 endurance athletes. This is highly relevant because athletes competing at the national, international, or world‐class level are exposed to very different training loads, race dynamics, metabolic demands, and adaptive ceilings than lower‐tier participants. A novice endurance athlete can improve performance primarily through training alone, making it difficult to isolate meaningful supplementation effects, whereas a Tier 3–5 athlete may benefit more from marginal gains during decisive race moments. Consequently, the external validity of the current evidence for highly trained endurance athletes remains limited, and practitioners should be cautious when extrapolating findings from lower‐level cohorts to elite contexts.

The characteristics of the event itself should also be considered before recommending either supplement. Creatine and beta‐alanine are more likely to be useful in endurance events with pacing variation, repeated accelerations, technical terrain, frequent climbs, or tactical variability, such as road cycling, triathlon, trail running, cross‐country events, some half‐marathons and marathons, and races decided by surges or final sprint capacity. In weight‐bearing disciplines, especially uphill‐dominant running events, concerns regarding creatine‐associated increases in body mass may still warrant individualized consideration, even if such effects are often overstated in the literature. Thus, event duration, elevation profile, terrain, pacing demands, and the timing and nature of decisive race segments should all inform supplementation decisions. There are also important clinical and practical considerations that should precede implementation. These include gastrointestinal tolerance, paraesthesia with beta‐alanine, adherence to multidose protocols, cumulative supplementation exposure, interaction with other evidence‐based supplements, body composition goals, and the athlete’s tolerance. Moreover, because many endurance athletes simultaneously use carbohydrate loading, caffeine, sodium bicarbonate, or nitrate strategies, it could be useful if future applied recommendations do not evaluate creatine or beta‐alanine in isolation only, but rather within the broader context of integrated sports nutrition planning.

Safety and tolerability should also be considered when implementing creatine and beta‐alanine supplementation in endurance athletes. Current evidence consistently supports the safety of long‐term creatine monohydrate supplementation when consumed at evidence‐based doses, with no clinically meaningful adverse effects reported in healthy individuals [46]. The most common practical consideration for beta‐alanine is paraesthesia, a transient and dose‐dependent sensation that can be minimized by dividing the total daily dose into smaller servings (typically ≤ 1.6 g per dose) or by using sustained‐release formulations [24]. From a practical perspective, adherence may represent an additional challenge for both supplements, as creatine requires sustained daily intake to maintain elevated intramuscular stores, while beta‐alanine supplementation generally requires several weeks of consistent intake and multiple daily doses before meaningful increases in muscle carnosine are achieved.

Future research should therefore adopt more ecologically valid study designs. Randomized controlled trials are needed specifically involving Tier 3–5 endurance athletes, including cyclists, triathletes, marathoners, trail runners, and ultraendurance competitors. Future studies should also evaluate supplementation effects during race‐relevant protocols for these athletes, such as repeated surges, uphill time trials, sprint finishes under fatigue, stochastic pacing simulations, and multiday training or racing blocks, rather than relying only on isolated VO_2_max or time‐to‐exhaustion tests. In addition, more studies are needed to examine the effects of creatine and beta‐alanine supplementation across different phases of periodized training, such as high‐intensity mesocycles, tapering periods, or competition preparation blocks. It will also be important for future investigations to stratify findings according to sex, training status, performance tier, event type, and body mass sensitivity, allowing for more individualized and clinically relevant conclusions. Research should explore whether chronic use of these supplements influences not only acute performance, but also durability, recovery, immune function, muscle damage, and adaptation across an entire season, especially in athletes exposed to high cumulative training stress.

It is important to interpret the available evidence with caution, as some findings are mechanistic or laboratory‐based, which should not be directly extrapolated to competitive endurance performance. Mechanistic and laboratory studies can establish biological plausibility but do not necessarily demonstrate real‐world efficacy. While studies demonstrating increases in phosphocreatine availability, skeletal muscle carnosine content, intracellular buffering capacity, glycogen storage, or attenuation of exercise‐induced inflammation provide strong physiological plausibility, these mechanisms do not necessarily translate into meaningful improvements in real‐world endurance competition. Likewise, improvements observed in laboratory‐based outcomes, such as VO_2_max, ventilatory threshold, time‐to‐exhaustion, or repeated sprint protocols, may not fully reflect the complex physiological, tactical, and environmental demands encountered in endurance races. The practical considerations discussed throughout this review are based on our interpretation of the current body of evidence, integrating findings from randomized controlled trials, mechanistic studies, and established physiological rationale.

In conclusion, creatine and beta‐alanine supplementation should not currently be viewed as universally beneficial ergogenic aids for all endurance athletes. Instead, their usefulness appears to depend on whether the athlete’s physiology, training structure, race demands, and performance goals create a meaningful opportunity for these supplements to exert their proposed effects. Beta‐alanine may be most relevant for athletes exposed to prolonged efforts near the severe‐intensity domain or frequent acidosis‐inducing race moments, whereas creatine may be particularly valuable when repeated high‐intensity bursts, sprint capacity, glycogen support, or recovery between efforts are performance‐limiting factors. The potential contribution of creatine and beta‐alanine to podium‐level performance in Tier 3–5 athletes is supported primarily by physiological plausibility and evidence from Tier 1‐2 athletes, rather than direct and definitive evidence. Well‐designed randomized controlled trials involving Tier 3–5 endurance athletes and race‐specific performance outcomes are needed before stronger practical recommendations can be made. For endurance athletes chasing the podium, these supplements may represent targeted, context‐specific tools within a broader performance strategy, but endurance‐specific evidence in these higher‐caliber athletes is still needed before more definitive practical recommendations can be made.

## Author Contributions

F.R.: conceptualization, methodology, supervision, writing–original draft, and writing–review and editing. S.C.F.: writing–review and editing, resources, and software. D.G.C.: writing–review and editing. E.C.: writing–review and editing. F.S.L.: writing–review and editing. C.K.: writing–review and editing. A.H.L.: investigation and writing–review and editing. J.C.R.N.: funding acquisition, supervision, and writing–review and editing.

## Funding

J.C.R.N. has been financially supported by Fundação de Amparo à Pesquisa do Estado de São Paulo (FAPESP) (2025/07056‐9).

## Conflicts of Interest

C.K. has received grants, honoraria, and consulting fees from companies who do business in areas related to exercise and nutrition. D.G.C. conducted industry‐sponsored research involving creatine supplementation and received creatine donations for scientific studies and travel support for presentations involving creatine supplementation at scientific conferences and serves on the Scientific Advisory Board for Alzchem, Create, and Iovate (companies that manufacture creatine products). S.C.F. is a scientific advisor for Bear Balanced and has received creatine donations from Creapure for research purposes. All disclosed relationships and collaborations had no influence on the literature search, interpretation of the evidence, or conclusions presented in this review. The manuscript was prepared independently, and the conclusions reflect the authors’ critical appraisal of the available scientific evidence. The other authors declare no conflicts of interest.
